# Adenomyoepithelial adenosis associated with breast cancer: a case report and review of the literature

**DOI:** 10.1186/2193-1801-2-50

**Published:** 2013-02-13

**Authors:** Hiroyuki Maeda, Shigehiro Yokoi, Masako Nakazawa, Kenji Koneri, Yoshiaki Imamura, Akio Yamaguchi

**Affiliations:** First Department of Surgery, Faculty of Medicine, University of Fukui, 23-3, Matsuoka, Shimoaizuki, Eiheiji-cho, Yoshida-gun, 910-1193 Fukui, Japan; Division of Surgical Pathology, University of Fukui Hospital, Fukui, Japan

**Keywords:** Adenomyoepithelial adenosis, Breast cancer, Adenomyoepithelioma, Fibrocystic disease

## Abstract

Adenomyoepithelial adenosis of the breast is an extremely rare type of adenosis. We herein present the case of a 35-year-old woman, who presented with a small painless hard lump and elastic soft induration of 5 cm in diameter in her left breast. Clinical examination and diagnostic workup were suggestive of a breast carcinoma, and a modified radical mastectomy and sentinel node biopsy were performed. Histopathological examination revealed adenomyoepithelial adenosis along with fibrocystic change and small invasive ductal carcinoma, slightly away from the adenosis. The presented case was thought to be initial-stage adenomyoepithelial adenosis and independently developing breast cancer. From a review of five reported cases of adenomyoepithelial adenosis, complete resection of the tumor and coexisting malignant disease may be recommended, owing to the tendency to develop breast cancer or malignant adenomyoepithelioma, or recurrence.

**Electronic supplementary material:**

The online version of this article (doi:10.1186/2193-1801-2-50) contains supplementary material, which is available to authorized users.

## Introduction

Adenomyoepithelial adenosis is an extremely rare type of adenosis associated with adenomyoepithelioma (Tavassoli & Soares^2003^; Moinfar^2007^) and has been shown to exhibit highly proliferative activity in both glandular epithelial and myoepithelial cells. This rare adenosis was considered to be prone to progression to definitive carcinoma (Tsuda et al.^1994^; Tavassoli^1991^). In this paper, the case of a 35-year-old woman with breast cancer combined with fibrocystic disease, which was diagnosed as adenomyoepithelial adenosis after mastectomy, is reported. Presenting symptoms, diagnostic evaluation and surgical management are discussed along with a review of the literature.

## Case presentation

**Figure 1:**
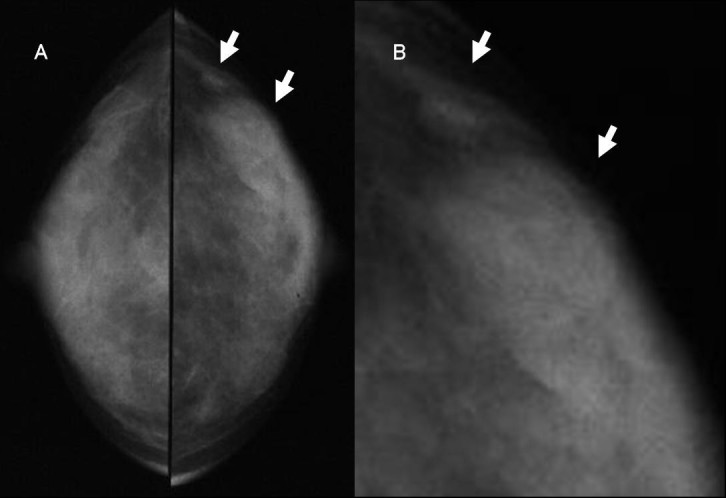
**A CC and B L-CC magnified view mammograms of the patient.** Focal asymmetric density with amorphous calcification and small mass with indistinct margins (arrows) are seen in the upper outer quadrant of the left breast.

**Figure 2:**
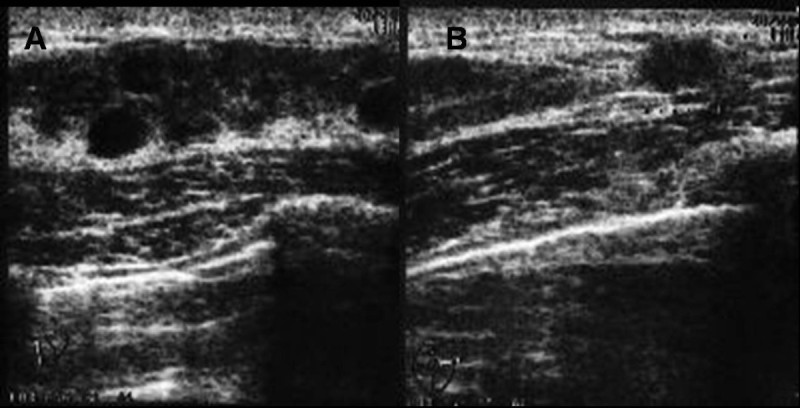
**A Ultrasonography of induration of the left breast demonstrates a low-echoic area measuring 5 × 5 × 1 cm with multiple cysts.B** Ultrasonography of the hard lump demonstrates an irregular hypoechoic mass measuring 0.6 × 0.6 × 0.4 cm near the low-echoic area described above.

**Figure 3:**
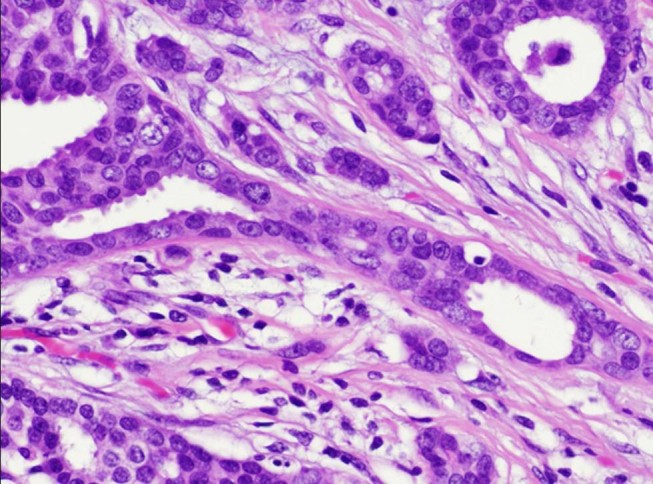
**Histological examination of the hard lump revealed a well-differentiated invasive ductal carcinoma (H&E stain, ×50).**

**Figure 4:**
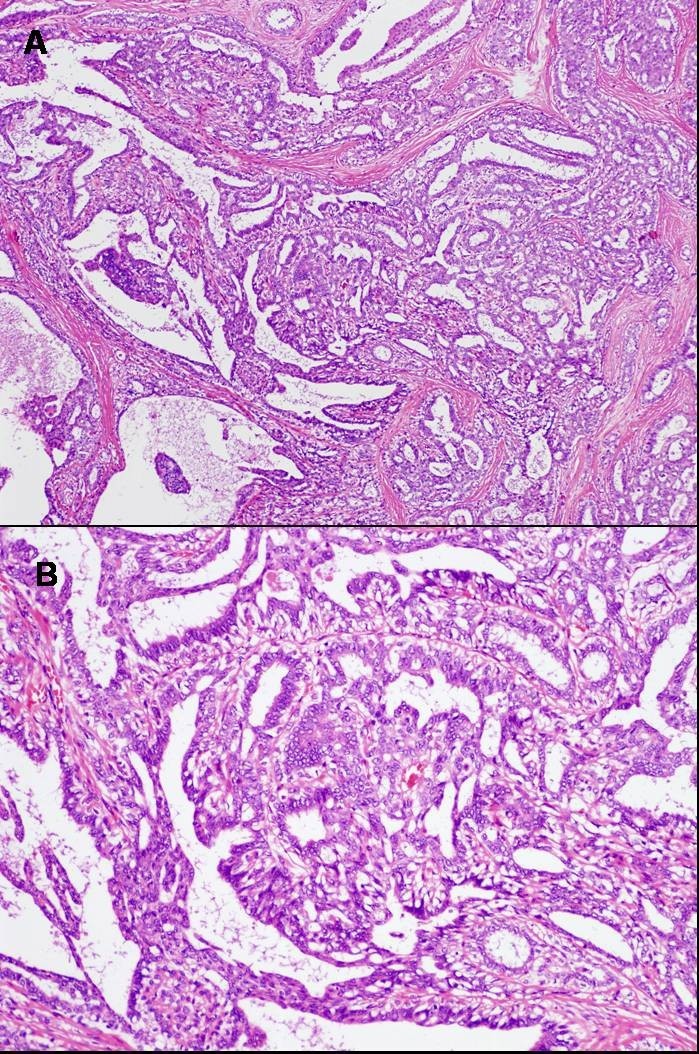
**Histological findings of adenomyoepithelial adenosis mixed with fibrocystic disease (H&E stain, A × 10, B × 25).**

**Figure 5:**
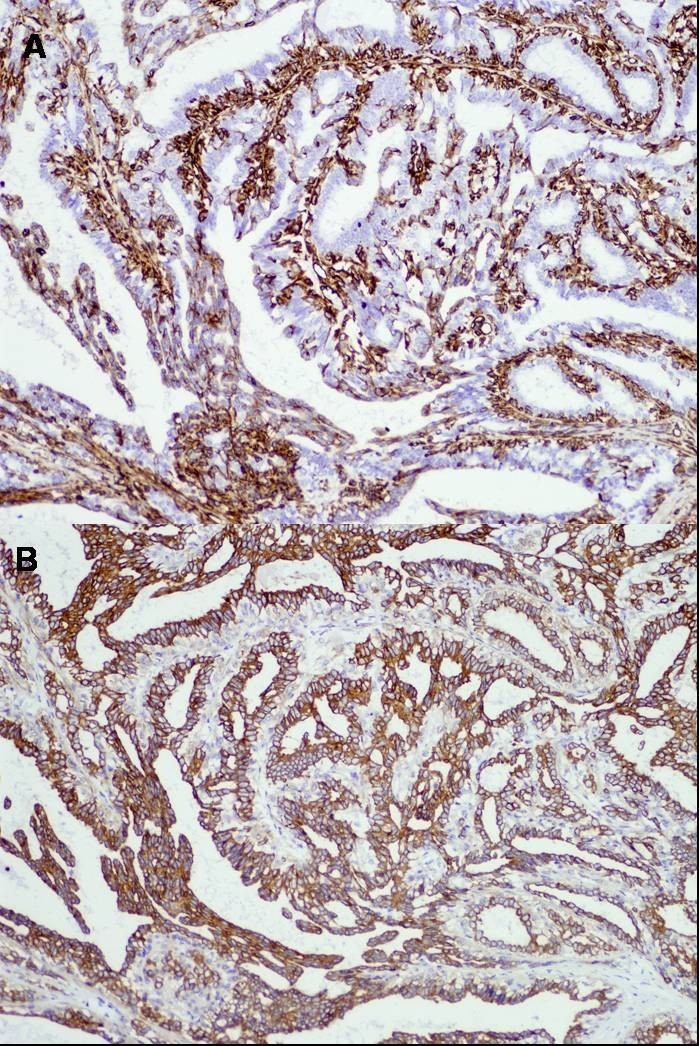
**Immunohistochemical localization of (A) vimentin and (B) cytokeratin AE1/AE3.** The myoepithelial cells were positive for vimentin (myoepithelial marker) and negative for cytokeratin AE1/AE3 (glandular cell marker).

This case involved a 35-year-old woman who had noticed a small nodule under the skin of the left breast. She did not have any personal or family history of cancer. On clinical examination, a hard lump measuring 0.7 × 0.6 cm was palpated in the upper outer quadrant of her left breast and also elastic soft induration approximately 5 × 4 cm was palpated in the outer quadrant of her ipsilateral breast. Axillary lymphadenopathy was absent. Mammography showed focal asymmetric density (FAD) in the outer portion of the left breast with segmental amorphous calcification in the outer portion of the left breast and an irregular small mass at the outside of FAD (Figure 1). Ultrasonography demonstrated an irregular hypoechoic mass measuring 0.6 × 0.6 × 0.4 cm and a low-echoic area measuring 5 × 5 × 1 cm including multiple small cysts inside the small mass (Figure 2). MRI showed rapid enhancement in the small mass and gradual enhancement in the inner area with multiple cysts. Routine blood and biochemical examinations as well as carcinoembryonic antigen (CEA) and carbohydrate antigen (CA15-3) were within normal limits. A core needle biopsy of these small nodules indicated invasive ductal carcinoma, and that of the soft induration indicated mastopathy. Preoperative staging with computed tomography scans and FDG-positron emission tomography revealed no distant metastasis. The patients subsequently underwent a left modified radical mastectomy and sentinel node biopsy, which was negative for metastasis. The cut surface of the induration lesion, 5 cm in size, revealed a glassy, whitish, indistinct lesion combined with multiple cysts, and that of the hard mass 0.6 cm in size showed yellowish tan-colored solid tumor with an irregular shape. Histological examination revealed a well-differentiated invasive ductal carcinoma with histological grade I according to the Bloom and Richardson modified classification on the small nodule (Figure 3). On immunohistochemical analysis, invasive ductal carcinoma cells were highly positive for both estrogen and progesterone receptors and negative for Her2 (score 1). On the other hand, the induration lesion was diagnosed as adenomyoepithelial adenosis along with fibrocystic changes. Microscopically, dilatation of the ducts, duct papillomatosis, sclerosing adenosis, duct ectasia and apocrine metaplasia were observed. Within the fibrocystic disease, there was an intraductal proliferation lesion, composed of hyperplasia of both myoepithelial cells and glandular epithelial cells. A bicellular pattern was preserved, and those myoepithelial cells had clear cytoplasm, with neither nuclear atypia nor cellular division (Figure 4A and4B). On immunohistochemical analysis, those myoepithelial cells were positive for basal cell or myoepithelial markers such as α-SMA, vimentin, HHF35, S-100 protein, CK5/6 and CK14, and negative for glandular cell markers such as cytokeratin AE1/AE3 (Figure 5A and5B). The patient underwent adjuvant endocrine therapy with tamoxifen at 20 mg/daily and is currently well 57 months after surgery. She did not desire breast reconstruction.

## Discussion

According to the WHO classification Tumors of the Breast and Female Genital Organs (Tavassoli & Soares^2003^) but not to the new WHO classification (Schmitt et al.^2012^), myoepithelial lesions of the breast are classified into myoepitheliosis, adenomyoepithelial adenosis, adenomyoepithelioma and malignant myoepithelioma. Adenomyoepithelial adenosis is an extremely rare type of adenosis associated with adenomyoepithelioma (Tavassoli & Soares^2003^; Moinfar^2007^). This adenosis consists of a diffuse proliferation of round or irregular tubular structures lined by a cuboidal to columnal epithelium, which may show apocrine metaplasia. There is a prominent, focally hyperplastic myoepithelial cell layer with strikingly clear cytoplasm. There is no significant nuclear atypia or mitotic activity. In most described cases, ademyoepithelial adenosis is mixed with or surrounds an adenomyoepithelioma (Tavassoli & Soares^2003^).

In terms of the histological findings of this case, hyperplasia of both glandular epithelial cells and myoepithelial cells that had extremely clear cytoplasm was observed, as shown in Figure 4 compatible with the findings of adenomyoepithelial adenosis as reported by Kiaer *et al.* (Kiaer et al.^1984^). On the other hand, the adenomyoepithelial adenosis in this case was mixed with fibrocystic changes such as dilated ducts, duct papillomatosis, duct adenosis, sclerosing adenosis, duct ectasia or apocrine metaplasia, but not with adenomyoepithelioma as previously reported in three cases (Kiaer et al.^1984^; Eusebi et al.^1987^; Eusebi et al. 1997). Although the reason for the difference in terms of coexisting disease was unknown, we speculated that this reported case was at the initial stage of such cases because of the very young age, lack of mass formation and the incidental finding after operation. The three previously reported cases were older than 50 years old and had a mass in their breast, suggesting that adenomyoepithelial adenosis may differentiate into benign or malignant adenomyoepithelioma with the passage of several decades (Kiaer et al.^1984^; Eusebi et al.^1987^; Eusebi et al.^1997^. Consistent with our speculation, Erel *et al.*(Erel et al.^2008^) reported a patient with adenomyoepithelial adenosis, who was 46 years old and presented with a palpable mass in her right breast. Excisional tumor was diagnosed as fibrocystic change and later, recurrent masses were palpable under the incisional scar. In the re-excisional biopsy specimen, histopathology showed adenomyoepithelial adenosis with neither significant atypia nor mitotic activity without breast cancer or adenomyoepithelioma.

From the viewpoint of Tsuda *et al.* (Tsuda et al.^1994^), adenomyoepithelial adenosis was considered to be prone to progression to adenocarcinoma of the breast. This is because they reported a 50-year-old patient with a breast tumor that was present for 20 years and increased in size to 5 cm. After mastectomy, the tumor was diagnosed as adenomyoepithelial adenosis, with highly proliferative activity in both glandular epithelial and myoepithelial cells, and five unmistakable adenocarcinoma foci under 1.0 cm in diameter. In our reported case, the tumor of adenocarcinoma, 0.7 cm in diameter, was slightly separate from the adenomyoepithelial adenosis, which showed neither significant atypia nor mitotic activity and was mixed with fibrocystic disease, suggesting that the occurrence of small breast cancer might be independent of the adenomyoepithelial adenosis.

From a review of five reported cases of adenomyoepithelial adenosis, all five cases had a palpable mass from 1.3 cm to 5 cm in size (Tsuda et al.^1994^; Kiaer et al.^1984^; Eusebi et al.^1987^; Erel et al.^2008^).

On mammography, one case had a speculated mass with pleomorphic calcification (Erel et al.^2008^), another case had a mass with irregular margin (Tsuda et al.^1994^), and a third case (Kiaer et al.^1984^) had focal asymmetric density. On ultrasonography, two cases showed an irregular hypoechoic mass with posterior acoustic shadow (Tsuda et al.^1994^; Erel et al.^2008^). The final diagnostic procedures for adenomyoepithelial adenosis were excisional biopsy in three cases (Kiaer et al.^1984^; Eusebi et al.^1987^; Erel et al.^2008^) and mastectomy in two cases (Tsuda et al.^1994^; Eusebi et al.^1987^), in one of whom aspiration cytology was considered false positive for carcinoma (Eusebi et al.^1987^). Our presented case showed induration of 5.5 cm in diameter and focal asymmetric density with microcalcification on mammography and a restricted hypoechoic area with small simple cysts on sonography; it was diagnosed after mastectomy. From these reported cases, preoperative diagnosis of adenomyoepithelial adenosis was thought to be difficult due to the lack of a specific finding on imaging or false-positive aspiration cytology. Core needle biopsy such as vacuum-assisted biopsy may be suitable (Yahara et al.^2008^), although this reported case failed to show adenosis, owing to combination with fibrocystic disease, using a 16-gauge needle.

Although it is difficult to reach a conclusion concerning the degree of malignancy, extirpation of the tumor may be recommended owing to the tendency to develop breast cancer or malignant adenomyoepithelioma (Tsuda et al.^1994^; Kiaer et al.^1984^), if the preoperative diagnosis of adenomyoepithelial adenosis is performed using needle biopsy. As two cases recurred after excisional biopsy (Kiaer et al.^1984^; Erel et al.^2008^), re-excision may be recommended in cases of suspected inadequate margin, like adenomyoepithelioma (Nadelman et al.^2006^). The best predictors of recurrence are initial incomplete or close excision margins. Therefore, correct preoperative diagnosis of the extent of such disease was thought to be important for surgical planning.

## Conclusion

We reported a case of adenomyoepithelial adenosis as an extremely rare type of adenosis, coexisting with invasive ductal carcinoma. Because the presented case was very young with no tumor formation and mixing with fibrocystic change, the case was thought to be an initial stage of adenomyoepithelial adenosis. Owing to the tendency to develop breast cancer or malignant adenomyoepithelioma and recurrence, complete resection of adenomyoepithelial adenosis may be recommended.

## Authors’ original submitted files for images

Below are the links to the authors’ original submitted files for images. Authors’ original file for figure 1 Authors’ original file for figure 2 Authors’ original file for figure 3 Authors’ original file for figure 4 Authors’ original file for figure 5
