# Supplementary figures and images for: Adenomyoepithelial adenosis associated with breast cancer: a case report and review of the literature
Source: Springerplus. 2013 Feb 13;2(1):50. doi: 10.1186/2193-1801-2-50 (PMC3581764; doi:10.1186/2193-1801-2-50)

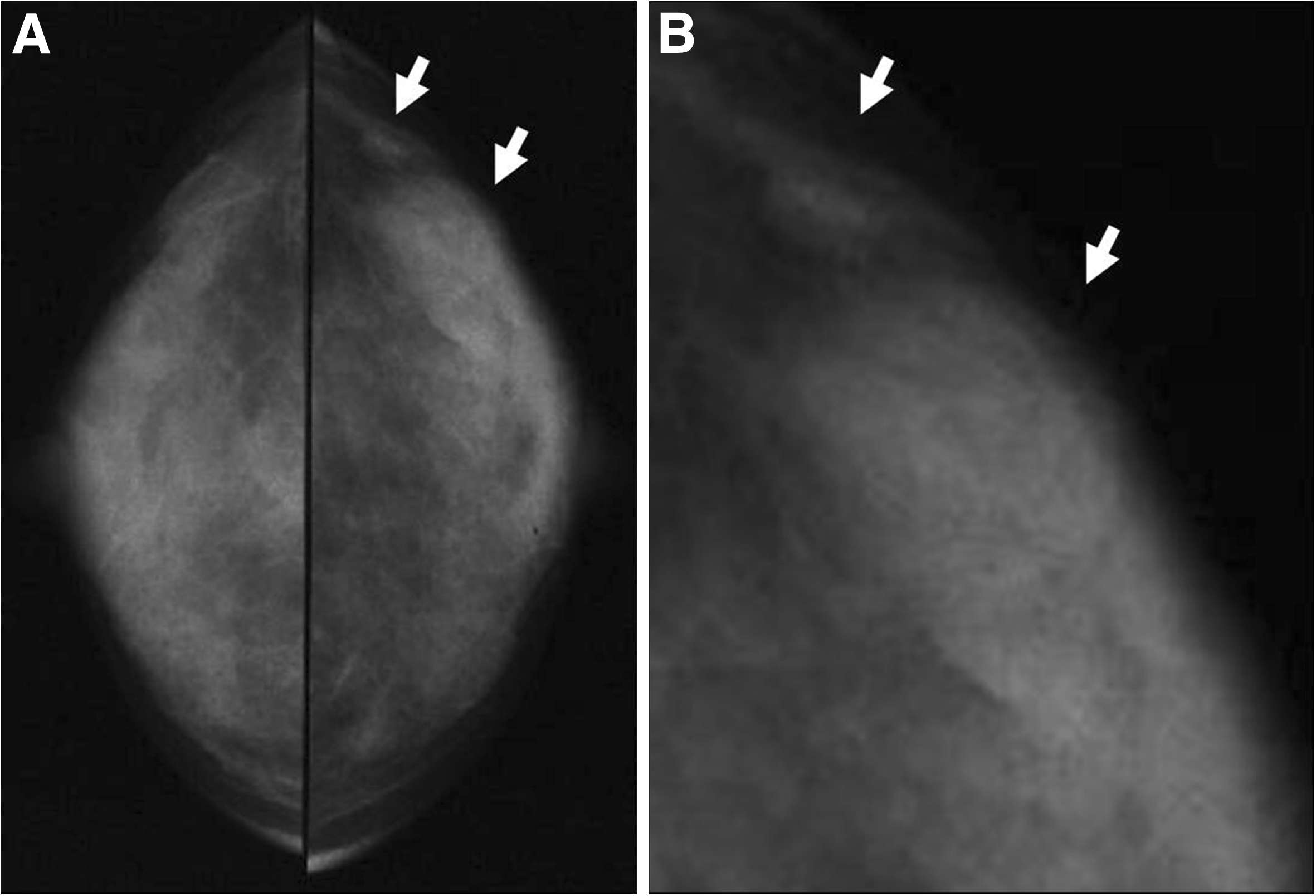

Supplement: Supplementary file 1 — Authors’ original file for figure 1 [file 40064_2013_91_MOESM1_ESM.tiff]

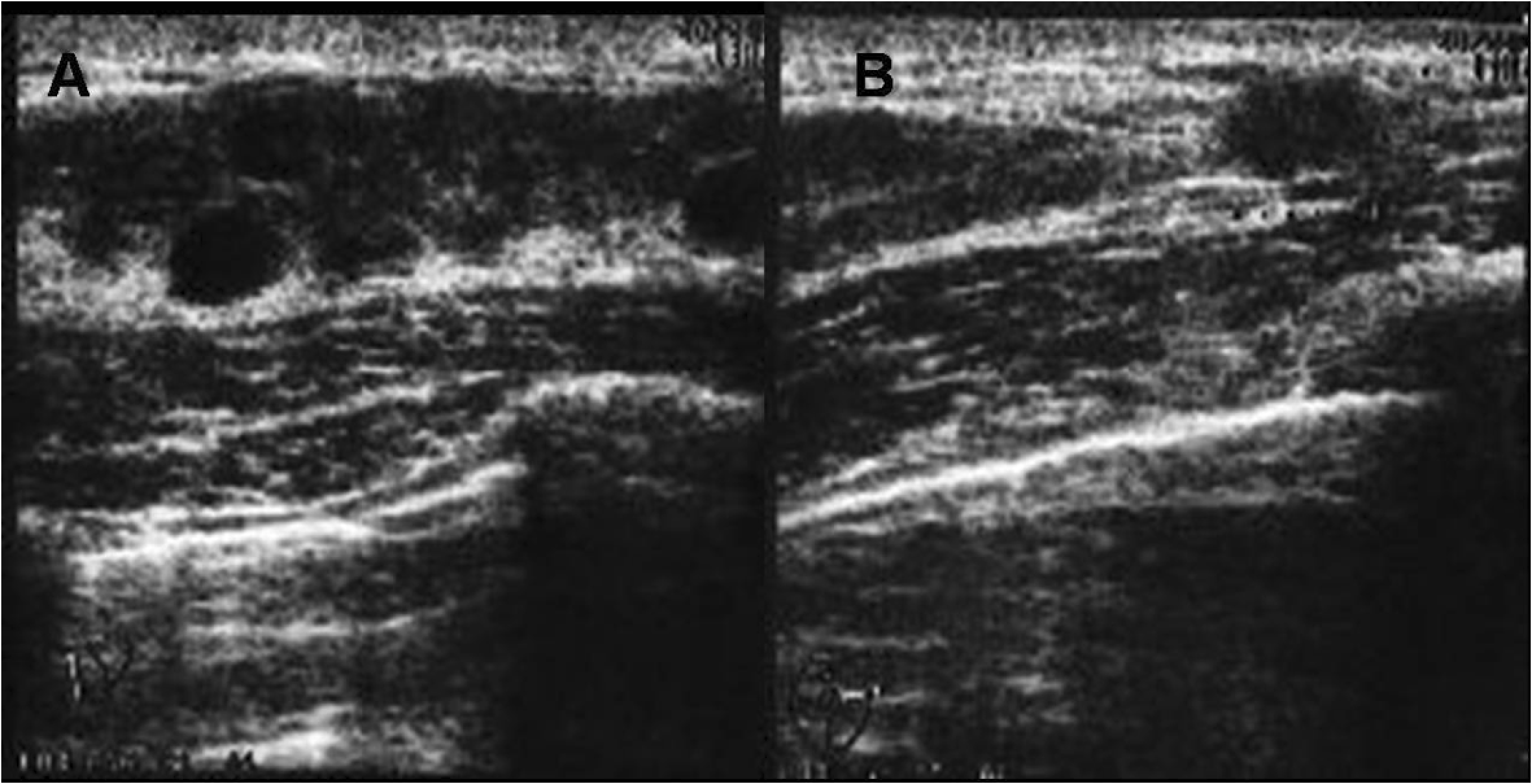

Supplement: Supplementary file 2 — Authors’ original file for figure 2 [file 40064_2013_91_MOESM2_ESM.tiff]

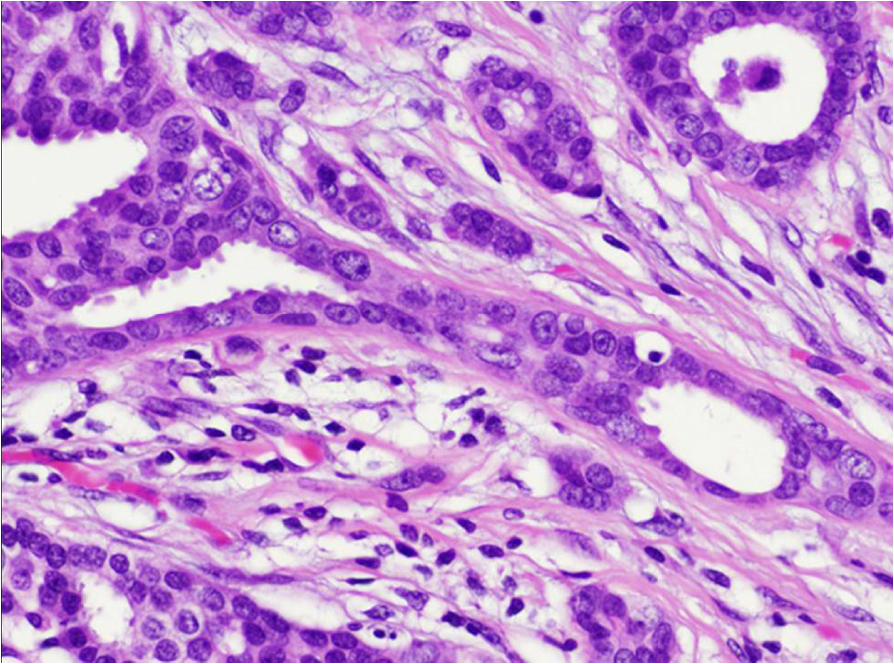

Supplement: Supplementary file 3 — Authors’ original file for figure 3 [file 40064_2013_91_MOESM3_ESM.tiff]

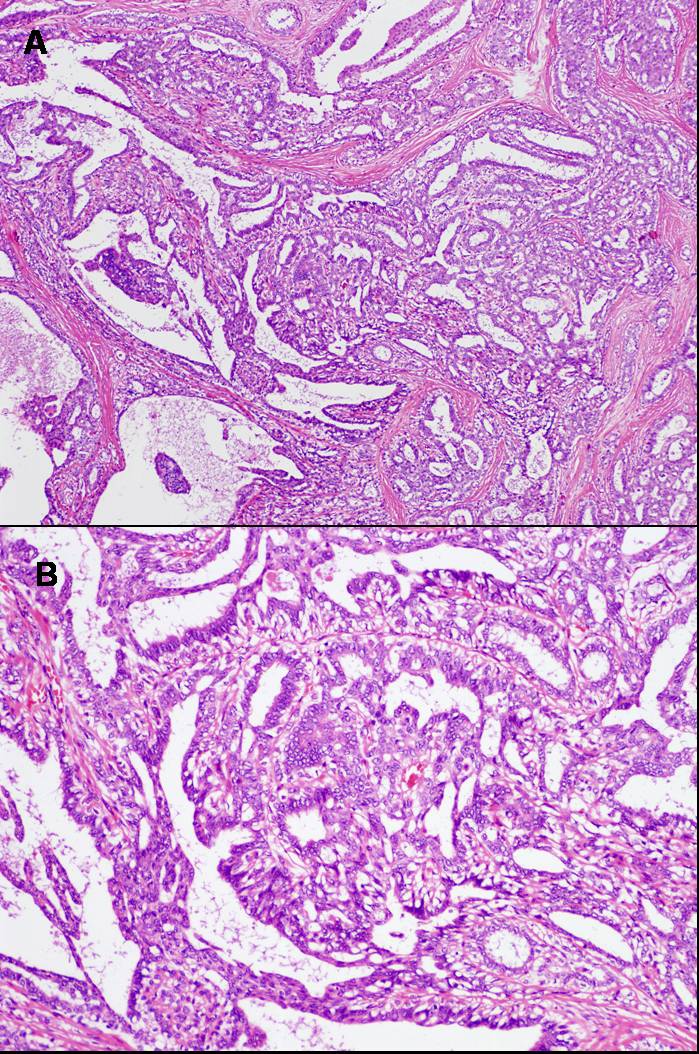

Supplement: Supplementary file 4 — Authors’ original file for figure 4 [file 40064_2013_91_MOESM4_ESM.jpeg]

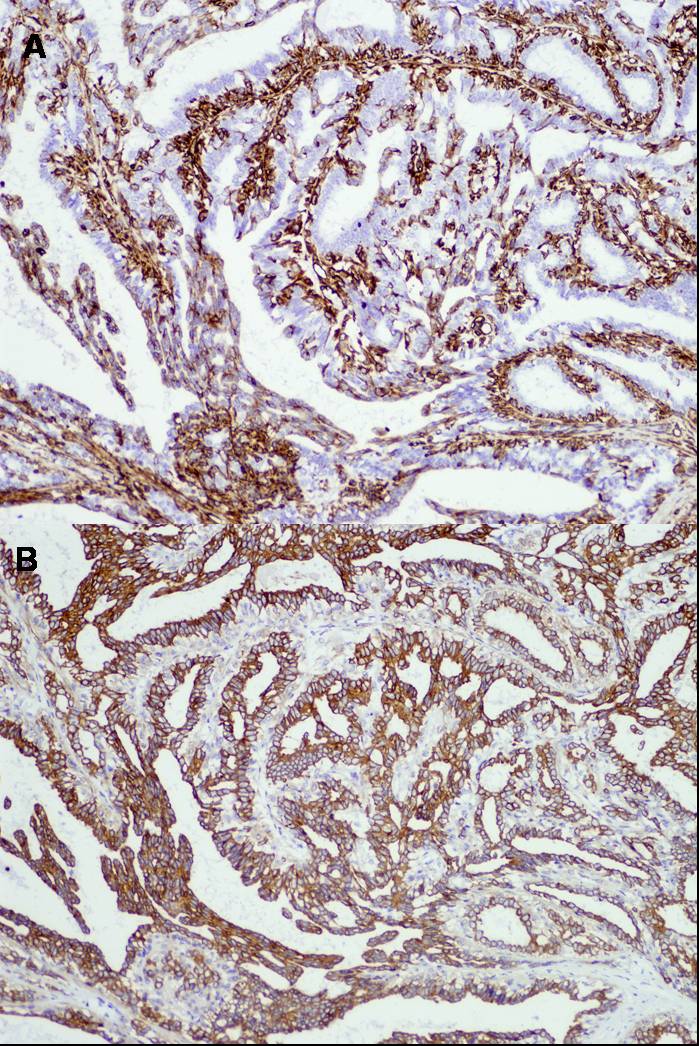

Supplement: Supplementary file 5 — Authors’ original file for figure 5 [file 40064_2013_91_MOESM5_ESM.jpeg]
